# 

**Published:** 1898-12

**Authors:** 


### The Library of the Bristol Medico-Chirurgical Society.

At the Annual Meeting, held on October 12th, 1898, Mr. L. M. Griffiths, the Honorary Librarian, read the following Report:—

The Library of the Bristol Medico-Chirurgical Society consists, including 671 duplicates, of 7,180 volumes.[1] During the year 1,360 volumes have been added, and 63 have been given to other Libraries or sold. The Library is in regular receipt of 170 periodicals.

The donors of books, since the last report, have been: The American Ophthalmological Society, Mr. Joshua S. Beddome, the British Balneological and Climatological Society, Mr. J. Paul Bush, Dr. W. F. Cleveland, the College of Physicians of Philadelphia, the Detroit Public Library, the Dundee Medical Library, Mr. T. F. Edgeworth, Messrs. James Fawn & Son, Mr. L. M. Griffiths, Mr. W. H. Harsant, Mr. C. B. Keetley, the Laryngological Society of London, Mrs. Henry Marshall, the Medical Society of London, the Medical Society of Pennsylvania, the Medical Society of the State of New York, the Family of the late Mr. J. S. Metford, the Staff of Middlesex Hospital, the Orleans Parish Medical Society, the Philadelphia County Medical Society, Dr. J. E. and Mr. A. W. Prichard, Dr. W. U. Reber, the Royal Academy of Medicine in Ireland,

[1] In the report printed in the *Journal* for December, 1897, for "5,833" read "5,883."

Mr. R. Sadler, Dr. J. E. Shaw, Mrs. Greig Smith, Dr. R. Shingleton Smith, the Trustees of the Smithsonian Institution, the Surgeon-General United States Army, and Messrs. J. Wright and Co. Framed pictures have been presented by Dr. A. J. Harrison, Dr. Aust Lawrence, Mrs. Henry Marshall, and Mrs. Greig Smith. Several persons have given unframed pictures and unbound periodicals.

A large number of medical portraits, obtained from various sources, have been framed in groups and are now on the walls. The Library has several duplicate portraits, which are offered for sale at little more than a nominal price.

Our Library advantages are greater than are represented in the ownership of the books belonging to this Society. By the constitution of the Bristol Medical Library, to which 2,088 volumes have been added during the year, we have the opportunity of consulting under this roof a collection of 18,750 volumes, contributed by the constituent Libraries in the following manner:

| | Volumes. |
|---|---|
| Bristol Medico-Chirurgical Society ... | 7,180 |
| Faculty of Medicine ... ... ... ... ... | 6,539 |
| Bristol Royal Infirmary ... ... ... ... | 3,650 |
| Bristol General Hospital ... ... ... | 1,381 |

In the conjoint Library 204 current periodicals are received.

The Library now possesses busts of William Budd and James Syme. Other busts or figures appropriate for a Medical Library would be most acceptable.

The following important complete sets of periodicals are now available for reference :—

*Annals of Surgery*, *Archives of Surgery*, *Asclepiad*, *Association Medical Journal*, *Braithwaite's Retrospect*, *British and Foreign Medical Review*, *British and Foreign Medico-Chirurgical Review*, *British Gynæcological Journal*, *British Journal of Dermatology*, *British Medical Journal*, *Centralblatt für Chirurgie*, *Centralbatt für Gynäkologie*, *Centralblatt für innere Medicin*, *Clinical Journal*, *Edinburgh Hospital Reports*, *Gould's Year-Book*, *Illustrated Medical News*, *Index Medicus*, *Journal of Experimental Medicine*, *Journal of Pathology and Bacteriology*, *Journal of Physiology*, *King's College Hospital Reports*, *Laboratory Reports of the Royal College of Physicians (Edinburgh)*, *Lancet*, *Liverpool Medico-Chirurgical Journal*, *London Medical Gazette*, *Medical Chronicle*, *Medical Times and Gazette*, *Medical Week*, *Practitioner*, *Quarterly Medical Journal*, *Ranking's Abstract*, *Reports and Papers of the American Public Health Association*, *Sajous's Annual*, *Schmidt's Jahrbücher* (since 1873), *St. Bartholomew's Hospital Reports*, *St. George's Hospital Reports*, *St. Thomas's Hospital Reports*, *Transactions of the American Association of Obstetricians and Gynecologists*, *Transactions of the American Ophthalmological Society* (since 1873), *Transactions of the American Surgical Association*, *Transactions of the Clinical Society of London*, *Transactions of the Royal Medical and Chirurgical Society of London*, *Transactions of the New York Academy of Medicine* (2nd Series), *Transactions of the Obstetrical Society of London*, *Transactions of the Ophthalmological Society of the United Kingdom*, *Transactions of the Pathological Society of London*, *Transactions of the Royal Academy of Medicine in Ireland*, *Transactions of the Sanitary Institute of Great Britain*, *Westminster Hospital Reports*.

The following important sets are nearly complete:—

*American Gynæcological and Obstetrical Journal*,[1] *American Journal of the Medical Sciences*,[2] *Dublin Journal of Medical Science*,[3] *Dublin Quarterly Journal of Medical Science*,[4] *Guy's Hospital Reports*,[5] *Johns Hopkins Hospital Bulletin*,[6] *Johns Hopkins Hospital Reports*,[7] *Journal of Cutaneous and Venereal Diseases*,[8] *Journal of the American Medical Association*,[9] *Provincial Medical and Surgical Journal*.[10]

Besides those which I have named there are several less important sets, some of which are complete and some very nearly so.

Considerable advance has been made with the work of cataloguing, and an increasing number of readers find the usefulness of the card-catalogue, the consultation of which has been made easier by the adoption of conspicuous subject-titles from the *Index Medicus* classification, and by the insertion of side-guides which give the first and second letters of all words which come first on the cards. In this connection, it is only just to acknowledge the excellent services rendered to the Library by the Clerk and Assistant-Clerk of the Medical School. The former for the whole time the conjoint Library has been in existence, now more than five years, and the latter for more than four years, have put into their work the best that was in them. The readiness and intelligence with which that work has been done have produced good fruit to the value of which I am sure all frequenters of the Library will be prepared to bear testimony.

As I shall in a short time be resigning my position as Librarian, I should like in this my last report to direct attention to one or two things which are of importance in connection with the work of the Library and its Librarian.

It is necessary to call to mind the circumstances which led to my present appointment. When the Library of the Bristol Medico-Chirurgical Society was founded in 1891 you appointed me your Librarian, and you have done me the honour of re-electing me to the post every year since. In July, 1893, your Library and the practically non-existent Library of the Medical School were united, and the management of the conjoint Library was vested in a Committee consisting of three members of the Faculty of Medicine and three members of this Society. I was one of the latter number. One duty of this Committee was to elect from their own body a Librarian to watch over the interests of the conjoint Library. For this office I was selected. There has never been a Librarian for the College section, and it would perhaps be inconvenient to have an acting Librarian for each portion of the Library, with pos-

[1] Wants Dec., 1891; June, 1892; Oct., 1897. [2] Wants N.S. No. 1, 1841; Nos. 3–7, 1841–42. [3] Wants Vols. LXIX.—LXXI., 1880–81. [4] Wants Nov., 1862, and Aug., 1867. [Since purchased.] [5] Wants Vol. XLVI., 1889. [This has now been given.] [6] Wants Vols. I. and II., 1889–91. [7] Wants Vol. I., 1889, and Nos. 1–4 of Vol. II., 1890. [8] Wants Vols. I. and II., 1883–84. [9] Wants July 11, Sept. 19, and 26, 1885. [10] Wants Vol. I. for 1840–41.

sibly a third Librarian distinct from the other two to look after the united Libraries. Anyone whom you elect as your Librarian would doubtless be willing to hold himself responsible for the welfare of the College section and for the whole Library in all its details. In any case it will be well for this Society to maintain the continuity of the office of Librarian, for before the conjoint scheme came into operation you had such an officer and the College section had not.

The Library to which you have access is no longer mainly an assortment of handbooks and monographs, to which the practitioner can turn for hints in a difficult or troublesome case, but is now a collection of medical literature of the first rank. With its plentiful list of periodicals current and in sets, its volumes of Transactions of most of the best societies and institutions, its special literature ancient and modern, its standard works of reference, it is a Library of which the medical profession in this neighbourhood should be proud. It owes its fame in large measure to the generosity of many donors, local and remote; to the assiduity of those who have been appointed on its Committee; and also to the willing and self-denying labours of the reviewers of the books sent to your *Journal*, who have had no reward for their work except the satisfaction of feeling that by it they have aided in forming a Library which has become useful in the highest degree to themselves and others.

But this large Library brings with it great responsibilities. A Library must be progressive, and this one ought to increase in importance even faster than it has hitherto done. Very soon the existing provision for books will be exhausted, and the authorities will then have to devise the best method for extension. It is a pity that those concerned in the original construction of this building were not more far-seeing than they were. An apartment of this size, if its daylight had been admitted solely through the roof, and the whole wall space devoted to book-shelves, with a gallery for its upper portion, would have been ample for many years. Some such alteration of structure will have to be considered at no very distant date. Many difficulties have already occurred, and it is right to mention here that this Society is indebted to the College authorities for the way in which these difficulties have been met.

The increasing Library has not only introduced problems concerning structure, it has also necessitated a change in the qualifications of its Librarian. No longer must he be one who has little more than a capacity for remembering the titles of the works under his care and an interest in the quantitative development of the Library. Now he must be a scholar, acquainted with several languages; a man of wide literary outlook, of comprehensive professional knowledge, of large bibliographical acquirements, gifted with the power of lucid exposition in order to make others realise the resources of their Library, and able to give demonstrations of the practical and historical nature of its grouped contents. In addition to all this, he must be prepared to give a large amount of time to the superintendence of the Library and its correspondence, and able from his position to get in touch with librarians in all parts of the world and from his knowledge to judge with accuracy in what particular respect advantage to his Library might be best secured. Also, to know how to render help to readers who turn to him for assistance must not be lost sight of among other qualifications of the Librarian, and even this must be exceeded if the Library is to give its best to the profession, for he must be ready, even before he is asked, to point students to the avenues by which they can reach the goal they are seeking.

I should like to be allowed to hold office till the end of the year, in order that the election of my successor may be made with deliberation, as it would be fatal to the best interests of this excellent Library if another Librarian of small capacity were appointed as its guardian.

On the eve of my departure from office, I should like gratefully to say that I am thankful for the privilege which was afforded me of having a considerable share in the initiation and progress of our modern Medical Library, and I offer my heartfelt and sincere thanks for the ready help that has been given to me by young and old, all of whom have shown a toleration of my shortcomings which will leave with me a fragrant memory certainly while this life lasts.

It has been an honourable position to have held, and it is one with which I would not lightly part; but I feel very strongly that although a person may have been able to find scope for the useful exercise of limited talents in the management of an institution, he should not allow personal ambition to hinder its advancing welfare when the occasion arises for different powers to be employed in its behalf.

---

*The following donations have been received since the publication of the list in September:*

*November 30th, 1898.*

| | |
|---|---|
| W. Johnstone Fyffe, M.D. (1) ... ... ... ... | 283 volumes. |
| L. M. Griffiths (2) ... ... ... ... ... ... ... | 3 " |
| Vaughan Harley, M.D. (3) ... ... ... ... ... | 2 " |
| W. H. Harsant (4) ... ... ... ... ... ... ... | 1 volume. |
| C. B. Keetley (5) ... ... ... ... ... ... ... | 1 " |
| F. Poole Lansdown (6) ... ... ... ... ... ... | 1 " |
| New York Academy of Medicine (7) ... ... ... | 4 volumes. |
| Medical Society of the State of New York (8) ... | 34 " |
| G. Parker, M.D. (9) ... ... ... ... ... ... ... | 1 volume. |

| | |
|---|---|
| Pathological Society of London (10) ... ... ... | 2 volumes. |
| B. M. H. Rogers, M.D. (11) ... ... ... ... ... | 1 volume. |
| R. Sadler (12) ... ... ... ... ... ... ... ... | 1 ,, |
| Mrs. Greig Smith (13) ... ... ... ... ... ... | 1 ,, |
| The Agent General for Tasmania (14) ... ... ... | 1 ,, |
| University College, Bristol (15) ... ... ... ... | 1 ,, |
| W. Roger Williams (16) ... ... ... ... ... ... | 6 volumes. |

Unbound periodicals have been received from Dr. George Parker. Mr. James Taylor has presented a framed picture, and the Editor of the *British Medical Journal* some unframed pictures.

### THIRTIETH LIST OF BOOKS.

The titles of books mentioned in previous lists are not repeated.

The figures in brackets refer to the figures after the names of the donors, and show by whom the volumes were presented. The books to which no such figures are attached have either been bought from the Library Fund or received through the *Journal*.

| | | |
|---|---|---|
| **Aitken, W.** ... | *Outlines of the Science and Practice of Medicine* ... (1) | 1874 |
| **Althaus, J.** ... | *A Treatise on Medical Electricity* ... ... (1) 2nd Ed. | 1870 |
| **Anderson, T. M'C.** | *Contributions to Clinical Medicine* (Ed. by J. Hinshelwood) ... ... ... ... ... ... ... ... ... ... | 1898 |
| *Army Hospital Corps, Manual of Instructions for Non-Commissioned Officers and Men of the* | ... ... ... ... ... ... ... (1) | 1875 |
| **Atkinson, T. R.** ... | *Aids to Examinations.* Part II. ... ... ... ... ... | [N.D.] |
| **Bartholow, R.** ... | *Materia Medica and Therapeutics* ... ... ... ... (1) | 1877 |
| **Beale, L. S.** ... | *Morality* ... ... ... ... ... ... ... ... ... ... (1) | 1887 |
| **Bell, R.** ... ... | *Chloroform: Its Absolutely Safe Administration* ... ... | 1898 |
| **Bergey, D. H.** ... | *On the Resistance of Animals to Micro-Organisms* ... | 1898 |
| **Binz, C.** ... ... | *The Elements of Therapeutics.* (Tr. from 5th German Ed., and Ed. by E. I. Sparks) ... ... ... (1) | 1877 |
| **Blake, E.** ... ... | *On the Hand* ... ... ... ... ... ... ... ... ... ... | [1898] |
| **Blanford, H. F.** | *The Climates and Weather of India, Ceylon, and Burmah* | 1889 |
| **Bramwell, B.** ... | *Diseases of the Heart* ... ... ... ... ... ... ... (1) | 1884 |
| ,, ... | *Practical Medicine and Medical Diagnosis* ... ... (1) | 1887 |
| **Bristowe, J. S.** ... | *Theory and Practice of Medicine* ... ... (1) 7th Ed. | 1890 |
| *British Association, 1898—Handbook to Bristol* | ... ... ... ... ... ... ... | 1898 |
| ,, ,, | ,, *Excursions* ... ... ... ... ... ... ... ... ... | 1898 |
| **Bullock, Rev. C.** [Ed.] | *The Temperance Witness-Box* ... ... ... ... (1) | [N.D.] |
| **Burr, C. B.** ... ... | *Psychology and Mental Disease* ... ... ... 2nd Ed. | 1898 |
| **Campbell, C. M.** ... | *The Skin Diseases of Infancy and Early Life* ... (1) | 1889 |
| **Churchill, F.** ... | *The Diseases of Children* ... ... ... ... (1) 2nd Ed. | 1858 |
| **Clouston, T. S.** ... | *Mental Diseases* ... ... ... ... ... ... ... 5th Ed. | 1898 |
| **Cokenower, J. W.** | *Contributions to Orthopedic Surgery* ... ... ... ... | 1898 |
| **Cooke, J. P.** ... | *Chemical Philosophy* ... ... ... (1) Revised Ed. | 1882 |
| **Corfield, W. H.** ... | *The Treatment and Utilisation of Sewage* ... 3rd Ed. | 1887 |
| **Cripps, H.** ... ... | *Ovariotomy and Abdominal Surgery* ... ... ... ... | 1898 |

| | | | |
|---|---|---|---|
| **DaCosta, J. C.** | *Modern Surgery* | [2nd] Ed. | 1898 |
| **Dalby, W. B.** | *Diseases and Injuries of the Ear* | (1) | 1873 |
| **Dowse, T. S.** | *Neuralgia* | (1) | 1880 |
| **Dukes, C.** | *School Diet* | (1) | 1891 |
| **Dunglison, R.** | *A Dictionary of Medical Science* | (1) Revised Ed. | [1857] |
| **Farquharson, R.** | *School Hygiene and Diseases Incidental to School Life* | (1) | 1885 |
| **Fenwick, S.** | *Medical Diagnosis.* (Ed. by B. Fenwick) | (1) 6th Ed. | 1886 |
| **Fothergill, J. M.** | *Indigestion and Biliousness* | (1) | 1881 |
| **Fürstner, C.** | *Fürsorge für Gemüthskranke* | | 1899 |
| **Fuller, H. W.** | *Diseases of the Lungs* | (1) 2nd Ed. | 1867 |
| **Gadd, H. W.** | *A Synopsis of the British Pharmacopœia, 1898* | [3rd Ed.] | 1898 |
| **Galabin, A. L.** | *Diseases of Women* | (1) 4th Ed. | 1887 |
| **Gibson, G. A.** | *Diseases of the Heart and Aorta* | | 1898 |
| **Gleason, E. B.** | *Essentials of the Diseases of the Ear* | 2nd Ed. | 1898 |
| **Guérin, A.** | *Élements de Chirurgie opératoire* | (1) | 1855 |
| **Habershon, S. O.** | *Diseases of the Stomach* | (1) 3rd Ed. | 1879 |
| **Haig, A.** | *Diet and Food in Relation to Strength and Power of Endurance* | | 1898 |
| **Hare, F. E.** | *The Cold-Bath Treatment of Typhoid Fever* | | 1898 |
| **Herz, H.** | *Die Störungen des Verdauungsapparates* | | 1898 |
| **Hillier, T.** | *Diseases of Children* | (1) | 1868 |
| **Homfray, C. T. Kingzett and D.** | *A Pocket Dictionary of Hygiene* | (11) | 1898 |
| **Hyde, J. N.** | *Diseases of the Skin* | (1) | 1883 |
| **Isenthal and H. S. Ward, A. W.** | *Practical Radiography* | 2nd Ed. | 1898 |
| **James, P.** | *Sore Throat* | (1) 4th Ed. | 1879 |
| **Jessop, W. H. H.** | *Ophthalmic Surgery and Medicine* | | 1898 |
| **Johnson, L.** | *A Medical Formulary* | (1) | 1882 |
| **Keetley, C. B.** | *Index of Surgery* | (1) | 1881 |
| **Kennedy, H.** | *On Fatty Heart* | (1) | 1880 |
| **Kingzett and D. Homfray, C. T.** | *A Pocket Dictionary of Hygiene* | (11) | 1898 |
| **Kirkes, W. S.** | *Hand-Book of Physiology.* 11th Ed. (Ed. by W. M. Baker and V. D. Harris) | (1) | 1884 |
| **Leaf, C. H.** | *The Surgical Anatomy of the Lymphatic Glands* | | 1898 |
| **Legg, J. W.** | *Bile Jaundice and Bilious Diseases* | (1) | 1880 |
| **Longmore, T.** | *Treatise on Ambulances* | (1) | [N.D.] |
| **M'Cosh, Rev. J.** | *The Intuitions of the Mind* | (1) | 1860 |
| **McFarland, J.** | *Pathogenic Bacteria* | 2nd Ed. | 1898 |
| **Mackenzie, M.** | *Diphtheria* | (1) | 1879 |
| **Maclagan, T. J.** | *Rheumatism* | (1) | 1881 |
| **Maddox, E. E.** | *The Ocular Muscles* | | 1898 |
| **Martin, Sir J. R.** | *The Influence of Tropical Climates* | (1) 2nd Ed. | 1861 |
| **Martindale, W.** | *Materia Medica* | 10th Ed. | 1898 |
| ,, **and W. W. Westcott.** | *The Extra Pharmacopœia* | (1) 3rd Ed. | 1884 |
| **Meigs and W. Pepper, J. F.** | *Diseases of Children* | (1) 7th Ed. | 1882 |
| **Milton, J. L.** | *Gonorrhœa* | (1) 5th Ed. | 1883 |
| **Mitchell, S. W.** | *Fat and Blood* | (1) 2nd Ed. | 1878 |
| **Morris, H.** | *On the Origin and Progress of Renal Surgery* | | 1898 |
| ,, [Ed.] | *A Treatise on Anatomy* | 2nd Ed. | 1898 |
| **Morris, M.** | *Diseases of the Skin* | New [2nd] Ed. | 1898 |
| ,, [Ed.] | *The Book of Health* | (1) | 1883 |

| | | |
|---|---|---|
| **Morton and F. Woodbury, T. G.** | *The History of the Pennsylvania Hospital, 1751—1895* .. ... ... (4) Revised [2nd] Ed. | 1897 |
| **Moure, E. J.** ... | *Des Adénoïdites chez les Adultes* ... ... ... ... ... ... | 1898 |
| ,, ... | *De la Trachéo-Thyrotomie dans le Cancer du Larynx* ... | 1898 |
| **Myers, A. B. R.** ... | *Diseases of the Heart among Soldiers* ... ... ... (1) | 1870 |
| **Napheys, G. H.**... | *Medical Therapeutics* ... ... ... ... (1) 6th Ed. | 1879 |
| **Nettleship, E.** ... | *Diseases of the Eye* ... ... ... ... ... ... (1) 3rd Ed. | 1884 |
| **Palmberg, A.** ... | *Public Health.* (Translated, and the Section on England Edited, by A. Newsholme) ... 2nd Ed. | 1895 |
| **Paterson, H. S.** ... | *Life, Function, Health* ... ... ... ... .. ... (1) | 1884 |
| **Pepper, J. F. Meigs and W.** | *Diseases of Children* ... ... (1) 7th Ed. | 1882 |
| **Piffard, H. G.** ... | *Materia Medica and Therapeutics of the Skin* ... (1) | 1881 |
| **Playfair, W. S.** ... | *The Science and Practice of Midwifery.* 2 vols. 9th Ed. | 1898 |
| **Poland, J.** ... ... | *Traumatic Separation of the Epiphyses* ... ... ... ... | 1898 |
| ,, ... ... | *Skiagraphic Atlas* ... ... ... ... ... ... ... ... ... | 1898 |
| **Poulton, E. B.** ... | *Charles Darwin and the Theory of Natural Selection* ... | 1896 |
| **Powell, R. D.** ... | *Pulmonary Consumption* ... ... ... ... ... ... ... (1) | 1872 |
| **Purdy, C. W.** ... | *Practical Uranalysis and Urinary Diagnosis* ... 4th Ed. | 1898 |
| **Ramsay, A. M.** ... | *Atlas of External Diseases of the Eye* ... ... ... ... | 1898 |
| **Ringer, S.** ... ... | *Handbook of Therapeutics* ... ... ... (1) 10th Ed. | 1883 |
| **Roberts, W.** ... | *On Urinary and Renal Diseases* ... ... (1) 4th Ed. | 1885 |
| **Sadler, R.** ... ... | *Malâkî : Rendered apart from the Original* ... ... (12) | 1898 |
| **Scudamore, C.** ... | *On Gout, Rheumatism, and Gravel* ... (1) 2nd Ed. | 1817 |
| **[Seeley, J. R.]** ... | *Natural Religion* ... ... ... ... ... ... (1) 2nd Ed. | 1882 |
| *Shaw's Manual of the Vaccination Law* ... ... ... ... ... ... 6th Ed. | | 1898 |
| **Sheild, A. M.** ... | *Diseases of the Breast* ... ... ... ... ... ... ... ... | 1898 |
| **Shenstone, W. A.** | *Justus von Liebig : his Life and Work (1803—1873)* ... | 1895 |
| **Slater and E. J. Spitta, C.** | *An Atlas of Bacteriology* ... ... ... ... ... | 1898 |
| **Smith, A.** ... ... | *Ringworm* ... ... ... ... ... ... ... ... (1) 3rd Ed. | 1885 |
| **Smith, E.** ... ... | *Manual for Medical Officers of Health* ... ... ... (1) | 1873 |
| **Smith, Rev. H. P.** [Ed.] | *Glossary of Terms and Phrases* ... ... ... (1) | 1883 |
| **Spigelius, A.** ... | Αγγειολογία*: or, A Description of the Vessells in the Body of Man* ... ... ... ... ... ... ... (9) | 1649 |
| **Spitta, C. Slater and E. J.** | *An Atlas of Bacteriology* ... ... ... ... ... | 1898 |
| **Squire, P.** ... ... | *Companion to the British Pharmacopœia.* 16th Ed. (Revised by P. W. and A. H. Squire.) (1) | 1894 |
| **Stroebe, H.** ... | *Ueber die Wirkung des neuen Tuberkulins TR auf Gewebe und Tuberkelbacillen* ... ... ... ... ... | 1898 |
| *Tasmania and its Mineral Wealth* ... ... ... ... ... ... ... ... (14) | | 1898 |
| **Taylor, F.** ... ... | *Practice of Medicine* ... ... ... ... ... 5th Ed. | 1898 |
| **Teale, T. P.** ... | *Dangers to Health* ... ... ... ... ... (1) 4th Ed. | 1883 |
| **Thorowgood, J. C.** | *Notes on Asthma* ... ... ... ... ... (1) 3rd Ed. | 1878 |
| **Tobin, R. F.** ... | *A Synopsis of Surgery* ... ... ... ... ... ... ... | 1898 |
| *Vaccination and its Results, based on the Evidence taken by the Royal Commission, 1889–97, A Report on.* Vol. I. ... .. | | 1898 |
| **Vierordt, O.** ... | *Medical Diagnosis.* (Tr. by F. H. Stuart) .. (1) | 1891 |
| **Ward, A. W. Isenthal and H. S.** | *Practical Radiography* ... ... 2nd Ed. | 1898 |
| **Waring, E. J.** ... | *A Manual of Practical Therapeutics* ... (1) 2nd Ed. | 1865 |
| **West, C.** ... ... | *Diseases of Infancy and Childhood* ... (1) 6th Ed. | 1874 |
| **West, S.** ... ... | *How to Examine the Chest* ... ... ... ... ... (1) | 1883 |
| **Westcott, W. Martindale and W. W.** | *The Extra Pharmacopœia* (1) 3rd Ed. | 1884 |

**Whitla, W.** ... *Pharmacy, Materia Medica, and Therapeutics* (1) 5th Ed. 1889
**Wise, A. T. T.** ... *How to Avoid Tubercle* ... ... ... ... ... ... ... 1898
**Woodbury, T. G. Morton and F.** *The History of the Pennslyvania Hospital, 1751—1895* ... ... ... (4) Revised [2nd] Ed. 1897
**Ziemssen, H. von.** [Ed.] *Cyclopædia of the Practice of Medicine.* (English Edition, Ed. by A. H. Buck) Vols. I.—XVII. (1) 1875-80

---

### TRANSACTIONS, REPORTS, JOURNALS, &c.

American Gynæcological and Obstetrical Journal, The Vols. V., VI., 1894-95, XII. 1898
American Surgical Association, Transactions of the Vols. III.—V., 1885-87, XVI. 1898
Annals of Military and Naval Surgery ... ... ... ... (1) Vol. I. 1864
Annals of Surgery ... ... ... ... ... ... ... ... ... Vol. XVI. 1892
Army Medical Department—Reports for 1859... ... ... ... ... (1) 1861
Association Française de Chirurgie ... ... ... ... ... XI$^{e}$ Congrès 1897
Association of American Physicians, Transactions of the Vol. XIII. 1898
Brain ... ... ... ... ... ... ... ... ... ... (1) Vols. II.—V. 1880-83
Buffalo Medical Journal ... ... ... ... ... ... ... ... Vol. XXXVII. 1898
Bulletin of the New York Academy of Medicine (7) Vols. I.—IV. 1862-71
Centralblatt für Chirurgie ... ... ... ... ... ... ... ... ... 1874-92
Cincinnati Lancet-Clinic, The ... ... ... ... ... ...Vol. LXXIX. 1898
Clinical Society's Transactions ... ... ... ... ... ... Vol. XXXI. 1898
Edinburgh Obstetrical Society, The Transactions of the Vol. XXIII. 1898
Grant College Medical Society, Transactions of the, 1897 ... ... ... 1898
Hospital, The ... ... ... ... ... ... ... ... ... ... Vol. XXIV. 1898
Hospitals—
 Guy's Hospital Reports ... ... ... ... ... ... (6) Vol. XLVI. 1889
 Middlesex Hospital, The—Registrars' Reports for 1870 ... (16) 1871
International Library Conference, 1897, Transactions of the Second ... 1898
Iowa State Medical Society, Transactions of the ... ... Vol. XVI. 1898
Journal of Experimental Medicine, The ... ... ... ... ... Vol. I. 1896
Lancet, The... ... ... ... ... ... ... ... ... ... ... ... ... (1) 1874-75
London Medical Record, The ... ... (1) Vols. II.—VI., 1874-78, X. 1882
Medical Chronicle, The ... ... ... ... ... ... ... ... N.S., Vol. IX. 1898
Medical Record ... ... ... ... ... ... ... ... ... ... Vol. LIII. 1898
Medical Society of London, Proceedings of the ... Vols. X.—XIV., 1887-91, XVI. 1893
New York Journal of Gynæcology and Obstetrics, The Vols. III., IV. 1893-94
New York, Transactions of the Medical Society of the State of (8) 1807-31 (reprinted in 1 vol., 1868), 1840-43 (reprinted in 1 vol., 1868), 1851, 1854, 1857, 1860, 1861, 1864, 1866, 1868-89; 1890; (8) 1891-93; 1894 ... ... ... ... ... ... ... ... ... 1898
Nordisk Kirurgisk Forenings, Forhandlinger ved 3:je Möde, 1897 1898
Ohio State Medical Society, Transactions of the ... ... ... ... .. 1898
Ophthalmological Society of the United Kingdom, Transactions of the Vol. XVIII. 1898
Pathological Report of University College, London ... (3) Vol. III. 1894
Pathological Society of London, Transactions of the (10) Vols. XLVIII., XLIX. 1897-98
Philadelphia Medical Journal, The ... ... ... ... ... ... ... Vol. I. 1898

| | | |
|---|---|---|
| Practitioner, The (1) Vols. VI.—XVI., 1871-76, XX.—XXV., 1878-80, XXX.—XXXV., 1883-85, XLVIII., XLIX. | | 1892 |
| Quarterly Medical Journal, The ... ... ... ... ... ... | Vol. VI. | 1898 |
| Retrospect of Medicine, Braithwaite's (1) Vols. XCIII.—XCV., 1886-87, | XCVIII. | 1889 |
| Royal Academy of Medicine in Ireland, Transactions of the | Vol. XVI. | 1898 |
| Scottish Medical and Surgical Journal, The ... ... ... ... | Vol. II. | 1898 |
| Society of Anæsthetists, The Transactions of the ... ... ... | Vol. I. | 1898 |
| South African Medical Journal, The ... ... ... ... ... | (13) Vol. I. | 1884 |
| South Carolina Medical Association, Transactions of the ... ... | | 1897-98 |
| Universal Medical Journal, The ... ... ... | N.S., Vols. IV., V. | 1896-97 |
| University College, Bristol—Calendars ... ... | (2) 1893-94; (15) | 1898-99 |
| University Medical Magazine... ... ... ... ... ... ... ... ... | Vol. X. | 1898 |
| West London Medico-Chirurgical Society, Proceedings of the | (5) Vol. III. | 1889 |

### Local Medical Notes.

### BRISTOL.

Health Report.—The Report for 1897 is the last one we shall have under the old city boundaries. In the next, the Medical Officers of Health will have to start afresh with a new area and absence of statistics to work on for the added districts.

Dr. Davies has given us this year an excellent report, and furnished us with some tables showing the "means" for the last, in some cases, sixty years, in others for a less period, of the death-rates and sickness. It would be impossible to give these in detail or even to criticise them at length. In some cases they are of great value, as in diphtheria; but in smallpox, for instance, the three epidemics of 1858, 1863-64, and 1872 so materially alter the mean, that what is really our average number of cases appears to be below the average.

Except for the epidemic of enteric fever in the autumn of last year, the health of the city has been excellent. The actual number of cases of each zymotic disease is lower in almost every instance than what it has been for several years, and the death-rate—17.17—is very satisfactory. We have again to congratulate a certain number of persons (386) who should be dead, according to averages, on the fact that they are still with us.

Two hundred and sixty-six cases were examined for diphtheria and in 106 the bacillus was found. Medical men evidently appreciate the work of Dr. Davies in this respect. A good deal of space is given to the epidemic of enteric fever, and the arguments that it was due to milk are very thoroughly gone into. We cannot here repeat the calculations made to show the possibility that it came from any other source, but can only say there was but one chance in 126 billions of its being due to any other cause. This is convincing enough, perhaps, even to the most active agitators who may be attempting to purify the Avon. We must refer our readers to the Report itself for further information on this point.

A short reference is made to the new Isolation Hospital, now nearing completion, at Ham Green, and we hope when it is ready for use to give a detailed description of the blocks, administration house and laundry.

The Report for 1897 is fully up to its fellows of previous years, and is of great value to all who take an interest in public health.

The *Port Sanitary District Report* states that only two cases of small-pox were found on ships entering the Port of Bristol, both occurring on board a ship which came from the Mediterranean. The Medical Officer has, however, been on the watch to guard us from the introduction of such diseases as the plague, cholera, and many more, and we are fortunate in seeing the yellow and black flag so seldom hoisted. The value of internotification between towns with Ports cannot be overestimated; and a passenger who had been on a troopship from Bombay, and had come to Bristol, was kept under observation for some time.

The public do not, we believe, know of the precautions which are taken to prevent the introduction of disease in this way, or of the constant inspection of ships entering the harbour that goes on night and day all the year round. During the year 1,645 ships were overhauled, and 282 only found to have sanitary defects, such as foul forecastles, defective ventilation or closets, leakages into crew's space, impure water-supply, etc. Most of the defects existed in foreign vessels, which means that our own shipowners are alive to the comforts and needs of the crews. It is interesting to know that this floating population was 16,384 persons. From personal experience we can testify to the excellent qualities of the "Luath."

During the year 101 canal boats were examined, with very satisfactory results.

---

Greig Smith Memorial.—On September 30th the final proceedings in connection with the Memorial were taken at the Bristol Royal Infirmary. It had been arranged that—after paying for the bust which had been placed in the Museum and Library, and the Medallion in the Infirmary operation rooms—the balance of the fund raised shall be handed to the Infirmary Committee towards the cost of the reconstruction of the operation room, in which Greig Smith was particularly interested.

A large number of persons attended a meeting held in a tent in the Infirmary Garden. Mr. F. R. Cross, the Ophthalmic Surgeon to the Infirmary and High Sheriff of the City, occupied the chair. He gave a brief history of the Infirmary, in the course of which he said that in 1745 there were 70 beds and 439 in-patients, while in 1895 there were 270 beds and 3,103 in-patients.

Mr. W. H. Thorp, the Architect, presented a report, in which he stated that the work had been carried out on the lines laid down by the late Mr. Greig Smith. The principal operating theatre measures 21 ft. 4 in. by 15 ft. 6 in., and is 26 ft. 6 in. high. At a height of about 8 ft. from the floor is a projecting gallery carried round three sides of the room, for the use of students. The walls, to a height of 15 ft., are lined with glazed tiles, the upper part ivory-white in colour, and the lower, up to the level of the floor of the students' gallery, of a light jade-green tint, with darker line borders of the same colour. The remainder of the walls and ceilings are plastered with Portland cement, and finished to a fine smooth surface with Parian cement, and the same applies to the other rooms of the department. They will eventually be painted with light-coloured paint, but at present are left as finished by the plasterer. Forming a centre-piece between two of the windows, the bronze medallion portrait of the late Mr. Greig Smith, modelled by Mr. John Fisher, has been fixed to the wall. The theatre is fitted up with specially-made white glazed sponge sinks, with wringers, lavatory basins with treadle action, slop sink, glass shelving supported by polished gun-metal brackets, and Berkefeld filters, &c. The small operating theatre, size 16 ft. 9 in. by 12 ft., and about 14 ft. high, has no students' gallery, the arrangements and fitments being similar to the larger theatre. The anæsthetic room, size 16 ft. 9 in. by 9 ft., and about 14 ft. high, is entered from the main corridor by means of wide folding doors, to admit of the ambulance, and has similar doors opening into each of the operating theatres. The surgeons' consulting room, which adjoins and opens out of the large operating theatre, has undergone little or no alteration, but, in common with the other rooms of the department, has been provided with electric light. The expenditure incurred in the work would probably amount to £2,700.

Sir William Mac Cormac then delivered the address which is printed on pages 301-7.

After the address, those present followed Sir William Mac Cormac and Mr. Cross to the operation room, which was unlocked by Sir William and declared open. Light refreshments were afterwards served in the Committee Room and the corridors.

All friends of the Bristol Royal Infirmary must join in congratulations to the Committee that they have been able to complete this renovation in so satisfactory a manner.

### Royal Medical Benevolent College.

The Council of Epsom College, of which Sir Joseph Fayrer is Chairman, has just sent out an appeal for further subscriptions. They say that unless they are better supported they shall be compelled to make a considerable reduction in their expenditure for charitable purposes. This Institution is one that every medical man should support. Where is the doctor who can say he couldn't find at least a guinea a year for such a purpose? The local Secretary for the College, Mr. L. M. Griffiths, 9 Gordon Road, Clifton, will gladly receive subscriptions.

### The College of Physicians of Philadelphia.

The next award of the Alvarenga Prize will be made on July 14, 1899. Essays intended for competition may be upon any subject in Medicine, and must be received by the Secretary of the College on or before May 1, 1899. Further information may be obtained from the Secretary.

LIST OF SUBSCRIBERS

TO

### The Bristol Medico-Chirurgical Journal.

*DECEMBER*, 1898.

---

### England.

### CHESHIRE.

NEW BRIGHTON.
A. W. Riddell

### CORNWALL.

EAST LOOE.
L. F. Houghton

FALMOUTH.
S. A. Lidiard

HELSTON.
B. C. Kendall

NEW QUAY.
A. Hardwick

PENZANCE.
W. S. Bennett
J. A. Fox
J. Symons

ST. AUSTELL.
W. Mason

### DEVONSHIRE.

BABBACOMBE.
T. Finch

BAMPTON.
A. R. Dorm

BARNSTAPLE.
C. H. Gamble
J. Harper
M. Jackson

BRIXHAM.
G. C. Searle

BROAD CLYST.
J. Somer

BUDLEIGH SALTERTON.
T. G. C. Evans
H. F. Semple

CHAGFORD.
A. D. Hunt

CHUDLEIGH.
C. L. Cunningham

CULLOMPTON.
G. G. Gidley

DARTMOUTH.
R. B. Searle

DEVONPORT.
F. E. Row

EXETER.
J. M. Ackland
H. Davy
C. Fenwick
R. V. Solly
E. B. Steele-Perkins
R. Thomas
L. H. Tosswill

HONITON.
T. W. Shortridge

ILFRACOMBE.
F. W. Langridge

MILLBROOK.
A. B. Cheves

MORTHOE.
E. J. Hollings

NEWTON ABBOT.
J. Culross

OTTERY ST. MARY.
W. A. H. Barrett

PAIGNTON.
J. Alexander

PLYMOUTH.
G. F. Aldous
R. H. Clay
Medical Society
E. B. Thomson
W. L. Woollcombe

PLYMPTON.
C. Aldridge
W. D Stamp

### DEVONSHIRE *(Continued).*

SOUTH MOLTON.
H. J. Smyth

TORQUAY.

H. Humphreys

W. Odell

R Pollard

W. Powell

Medical Society

C. H. Wade

WINKLEIGH.
J. H. Norman

### DORSETSHIRE.

DORCHESTER.
P. W. MacDonald

STURMINSTER NEWTON.

J. C. Leach

WEYMOUTH.
J. M. Lawrie

WIMBORNE.
A. J. H. Crespi

### ESSEX.

NEWPORT.
W. A. Smith

### GLOUCESTERSHIRE.

ALMONDSBURY.
D. G. Gerrish
J. C. MacWatters

BRISTOL.
W. R. Ackland
J. Ambrose
C. Atchley
T. R. Atkinson
W. M. Barclay
T. G. L. Baretti
B. J. Baron
A. E. Blacker
A. F. Blagg
E. C. Board
C. W. J. Brasher
H. F. Briggs
J. Broom
F. St. J. Bullen
E. F. H. Burroughs
J. P. Bush
A. Carr
T. M. Carter
W. F. Carter
T. Carwardine
R. H. Chilton
J. M. Clarke
T. V. Coker
H. Cook
W. H. Cory
W. Cotton
W. Coulter
F. R. Cross
J. Dacre
D. S. Davies
N. C. Dobson
E. L. W. Dunbar
E. Eberle
F. H. Edgeworth
W. Elder
C. Elliott
J. Ewens
E. Fawcett
R. G. Fendick
J. L. Firth
T. Fisher
A. L. Flemming
E. L. Fox
J. Freeman
W. J. Fyffe
E. B. Garland
T. T. Genge
W. C. Gent
A. N. G. Gibbs
W. G. Grace
J. S. Griffiths
L. M. Griffiths
W. B. T. Gubbin
C. D. G. Hailes
A. H. Haines
H. E. Harris
A. J. Harrison
W. H. Harsant
A. G. Hayman
C. A. Hayman
J. C. Heaven
C. K. C. Herapath
H. E. Hetling
H. Hill
T. Hill
General Hospital
G. A. Imlay
H. W. Kendall
F. P. Lansdown
R. G. P. Lansdown
A. E. A. Lawrence
E. L. Lees
R. C. Leonard
W. Ligertwood
J. J. S. Lucas
P. W. McCrea
G. Metcalfe
H. F. Mole
A. T. Morgan
C. A. Morton
G. T. Myles
B. G. Neale
W. N. Nevill
C. Newman
W. H. C. Newnham
T. D. Nicholson
C. O'Brien
A. Ogilvy
G. S. Page
G. Parker
J. H. Parry
J. St. J. G. Parsons
A. W. Peake
H. C. Pearson
W. A. Perry
C. M. Phillips
C. F. Pickering
W. E. Pountney
J. J. Powell
A. W. Prichard
J. E. Prichard
A. B. Prowse
B. M. H. Rogers
F. Rose
W. B. Roué
C. K. Rudge
H. T. Rudge
J. E. Shaw
E. J. Sheppard
E. M. Skerritt
G M. Smith
R. S. Smith
E. H. E. Stack
W. H. Steel
C. Steele
J. B. Stephenson
W. H. Stevens
F. W. Stoddart
J. Swain
J. G. Swayne
S. H. Swayne
W. C. Swayne
J. O. Symes
J. Taylor
W. J. Tivy
H. Waldo

### GLOUCESTERSHIRE *(Continued).*

BRISTOL *(continued).*

C. H. Walker
C. H. Wallace
J. H. Wathen
C. J. Whitby
J. Wilding
C. Williams
P. W. Williams
R. Williams
H. W. Windsor-Aubrey
C. Wintle

CHELTENHAM.

G. A. Peake
E. Trevithick
L. Winterbotham

CHIPPING SODBURY.

A. Grace
F. Fox

CIRENCESTER.

W. R. Cossham
O. H. Fowler
C. Mackinnon

COLEFORD.

L. B. Trotter

DOWNEND.

H. Skelton

FISHPONDS.

W. Brown

GLOUCESTER

R. W. Batten
W. R. Hadwen
J. G. Soutar

HAMBROOK.

E. Crossman
F. W. Crossman
W. F. B. Eadon

KINGSWOOD.

R. W. Brimacombe

LYDNEY.

W. P. Kennedy

PARKEND.

W. C. Halpin

REDFIELD.

J. W. Rodgers

STAPLETON.

H. A. Benham

STOKE BISHOP.

H. F. Parsons.
C. E. Wedmore

STONEHOUSE.

G. T. B. Watters

STOW-ON-THE-WOLD.

E. Dening

STROUD.

A. S. Cooke

TETBURY.

W. Wickham

THORNBURY.

E. M. Grace
L. H. Williams

WESTBURY-ON-TRYM.

A. Harvey
H. L. Ormerod

WINTERBOURNE

R. Eager
W. Eager

WOTTON-UNDER-EDGE.

T. C. Boyce
D. H. Forty

### HAMPSHIRE.

ALTON.

W. L. P. Bevan

BOURNEMOUTH.

J. S. Dickie
J. G. Harsant

CARISBROOKE.

J. Groves

FLEET.

H. Wilcox

LYMINGTON.

J. Rendall

SOUTHAMPTON

W. R. Y. Ives

SOUTHSEA.

J. W. Cousins

### HUNTINGDONSHIRE.

ST. NEOT'S.

T. C. Grey
J. H. Hemming

### KENT.

TUNBRIDGE WELLS.

W. J. K. Millard

### LANCASHIRE.

LIVERPOOL.

G. A. Hawkins-Ambler
Medical Institution

PRESTON

J. E. Garner

### MIDDLESEX.

### LONDON.

J. Berry
J. F. Evans
T. C. Fox
E. T. Hale
M. Handfield-Jones
W. H. A. Jacobson
H. M. Jones
Lord Lister
E. J. Maclean
J. Macready
A. L. Marshall
F. T. Roberts
J. Scott
W. J. Seward
E. C. Taylor
G. S. Woodhead

### TOTTENHAM.

W. H. Plaister

### MONMOUTHSHIRE.

### CALDICOT.

C. Corben

### NEWPORT.

W. Basset
C. B. Gratte
O. E. B. Marsh
C. Matthews
A. G. Thomas
H. E. Williams

### PONTYPOOL.

J. R. Essex
S. B. Mason

### RHYMNEY.

T. H. Redwood

### RISCA.

G. B. Robathan

### TREDEGAR.

G. A. Brown

### NORFOLK.

### WALPOLE ST. ANDREWS.

R. Smith

### NOTTINGHAMSHIRE.

### NOTTINGHAM.

C. B. Taylor
W. M. Willis

### OXFORDSHIRE.

### OXFORD.

H. P. Symonds

### SOMERSETSHIRE

### BATH.

G. A. Bannatyne
E. J. Cave
C. S. W. Cobbold
T. Cole
F. S. Cowan
S. Craddock
W. M. Ellis
A. E. W. Fox
H. F. A. Goodridge
T. B. Goss
P. King
F. Lace
T. P. Lowe
W. S. Melsome
S. E. Rigg
R. J. H. Scott
L. H. Walsh
L. A. Weatherly
J. Wigmore

### BLAGDON.

T. O. Halliwell

### BRIDGWATER.

F. J. C. Parsons
J. L. K. Pringle
R. H. F. Routh

### BRISLINGTON.

B. B. Fox

### BURNHAM.

F. C. Berry

### CHEDDAR.

R. W. Statham

### CLEVEDON.

H. J. Capron
T. Davis
W. J. Hill
S. Skinner
J. Martin

### CREWKERNE.

W. J. Penny
W. W. Webber

### CURRY RIVEL.

R. H. Vereker

### FRESHFORD.

C. E. S. Flemming

### FROME.

A. W. Dalby
J. M. Rattray

### ILMINSTER.

C. Munden

### KEYNSHAM.

C. Harrison
G. G. D. Willett

### SOMERSETSHIRE *(Continued).*

LANGPORT.

J. Morgan

LEIGH WOODS.
J. Harvey.

MIDSOMER NORTON.
A. Waugh
A. H. Whicher

MILVERTON.
C. Randolph

NORTH PETHERTON.
C. F. Hawkins

PILL.
T. W. S. Morgan

PORTISHEAD.
G. Boyd
W. Monckton
C. A. Wigan

SHEPTON MALLET.
J. T. Hyatt

SOUTH PETHERTON.

S. W. MacIlwaine

TAUNTON.
H. T. S. Aveline
Taunton Hospital
H. T. Rutherford

TIMSBURY.
F. Woods

WEDMORE.
J. Ford
R. P. Tyley

WELLINGTON.
J. Meredith

WELLS.

W. Fairbanks

W. A. L. Smith

WESTON-SUPER-MARE.
H. S. Ballance
C. P. Crouch
G. B. Fraser
E. F. Martin
G. F. Rossiter
R. Roxburgh
J. W. Smith
G. H. Temple
J. Wallace

WORLE.
F. St. J. Kemm

WRINGTON.
H. C. Bristowe
H. W. Collins

YEOVIL.
W. R. M. Semple

### SUFFOLK.

BECCLES.
F. H. Hudson.

### SURREY.

REDHILL.
C. M. Jessop

FARNHAM.
P. T. Tolputt

VIRGINIA WATER.
S. R. Philipps

### WARWICKSHIRE.

LEAMINGTON.
A. W. W. Hoffman

### WILTSHIRE.

BRADFORD-ON-AVON.
W. J. A. Adye
J. Beddoe

CALNE.
W. S. Batten

DEVIZES.
C. Eddowes

FOVANT.
C. Clay

MALMESBURY.
R. Kinneir

MERE.
J. McElfatrick

SALISBURY.
C. H. Ackland

SWINDON.
W. Howse
J. C. Maclean

WARMINSTER.
R. L. Willcox

### Scotland.

### ARGYLESHIRE.

OBAN.

H. R. McDonald

### EDINBURGH.

EDINBURGH.

C. A. L. Brough

### Ireland.

### ANTRIM.

BELFAST.

J. W. Browne

A. Jamison

J. A. Lindsay

### Wales.

### BRECONSHIRE.

TALGARTH.

W. Howells

### CARMARTHENSHIRE.

FERRYSIDE.

P. Williams

LLANELLY.

S. J. Roderick

### CARNARVONSHIRE.

ST. CLEARS.

R. Thomas

### GLAMORGANSHIRE.

ABERDARE.

E. Jones

CARDIFF.

P. R. Griffiths
T. H. Horder
D. R. Paterson
J. A. Phillips
W. Price
A. Sheen
Medical Society
J. L. Thomas
H. R. Vachell
J. Williams

GORSEINON.

T. Mitchell

MAESTEG.

W. H. Thomas

MERTHYR TYDVIL.

W. W. Jones
C. E. G. Simons
J. L. W. Ward

PENARTH.

R. F. Nell

PORTH.

H. N. Davies

SWANSEA.

E. Davies
R. C. Elsworth
E. Le C. Lancaster

TREHARRIS.

W. W. Leigh

### PEMBROKESHIRE.

HAVERFORDWEST.

E. P. Phillips

### Belgium.

COURTRAI.

Dr. Lauwers

### Italy.

GENOA.

S. Biamonti

### Switzerland.

DAVOS-PLATZ.

W. R. Huggard

### Egypt.

ALEXANDRIA.

J. Mackie

CAIRO.

M. A. Ruffer

### Cape Colony.

FORT BEAUFORT.

J. Shaw

### New Zealand.

LOWER HUTT.

J. R. Purdy

### United States.

COLORADO.

S. E. Solly

CHICAGO.

Columbia Medical Journal

### India.

H. J. Pocock

BHAGELPORE.

N. Muzumider

### INDEX.

Abdominal operations, Fifty consecutive intra-—Dr. J. Swain, 211.
Abdominal sections, Notes on thirty-two consecutive—Dr. J. M. Lawrie (*Review*), 350.
Abdominal surgery—J. G. Smith (*Review*), 154.
Adénoïdes, Résultats de l'examen histologique de 64 végétations—Dr. E. J. Moure (*Review*), 77.
Adénoïdites chez les adultes, Des—Dr. E. J. Moure (*Review*), 353.
Air, food, and exercises—Dr. A. Rabagliati (*Review*), 264.
Aldersmith, Dr. H.—Ringworm and alopecia areata (*Review*), 260.
Allbutt, Dr. T. C. [Ed.]—A system of medicine (*Review*), 62.
Alopecia areata, Ringworm and—Dr. H. Aldersmith (*Review*), 260.
American Gynecological Society. Transactions of the (*Review*), 172,
American Laryngological Association, Transactions of the (*Reviews*), 274, 359.
American Ophthalmological Society, Transactions of the (*Reviews*), 78, 358.
American Pediatric Society, Transactions of the (*Review*), 273.
American Physicians, Transactions of the Association of (*Review*), 271.
American Public Health Association, Reports and papers of the (*Review*), 357.
American Surgical Association, Transactions of the (*Review*), 357.
American year-book of medicine and surgery (*Review*), 173.
Anatomy, Human—Drs. J. Cleland and J. Y. Mackay (*Review*), 148.
Anatomy, Mammalian — Dr. H. Jayne (*Review*), 332.
Anders, Dr. J. M.—A text-book of the practice of medicine (*Review*), 251.
Anderson, Dr. I.—Yellow fever in the West Indies (*Review*), 349.
Anderson, W.—The deformities of the fingers and toes (*Review*), 158.
Aneurysms of the aorta—Dr. O. A. Browne (*Review*), 349.
Annales de la Société Belge de Chirurgie (*Review*), 275.
Annual and analytical cyclopædia of practical medicine (*Reviews*), 81, 261.
Anomalies and curiosities of medicine—Drs. G. M. Gould and W. L. Pyle (*Review*), 55.
Aorta, Aneurysms of the—Dr. O. A. Browne (*Review*), 349.
Aphasia and the cerebral speech mechanism—Dr. W. Elder (*Review*), 68.
Archives provinciales de chirurgie (*Review*), 81.
Archiv für verdauungs-krankheiten (*Review*), 362.
Asepsis der hände, Die chirurgische—Dr. W. Poten (*Review*), 76.

Baby, Our—Mrs. L. Hewer (*Review*), 168.
Bacteriology, Manual of—Drs. R. Muir and J. Ritchie (*Review*), 165.
Balfour, Dr. G. W.—Clinical lectures on diseases of the heart and aorta (*Review*), 333.
Ball, Dr. J. B.—A handbook of diseases of the nose and pharynx (*Review*), 169.
Barclay, W. M.—Report on surgery, 45.
Baron, Dr. B. J.—Report on laryngology and rhinology, 322.
Bashore, Dr. H. B.—Outlines of rural hygiene (*Review*), 353.
Bath as a health resort (*Review*), 269.
Bathing places, Bradshaw's dictionary of (*Review*), 269.
Beevor, Dr. C. E.—Diseases of the nervous system (*Review*), 337.
Berlin, Gynæcology in — Dr. H. Macnaughton-Jones (*Review*), 168.
Bice, Dr. G. D. Crothers and H. H. —Elements of Latin (*Review*), 346.
Bishop, Dr. S. S.—Diseases of the ear, nose, and throat, and their accessory cavities (*Review*), 73.
Black death, 96.
Bladder and urinary fever, Inflammation of the—Dr. C. M. Moullin (*Review*), 339.
Bladder, Illumination of the male, 238.

Blomfield, Obituary notice of Dr. A. G., 194.
Bramwell, Dr. B.—Diseases of the spinal cord (*Review*), 154.
Bristol health report, 378.
Bristol, Medical Bits of Old, 96, 196, 283.
Bristol Medico-Chirurgical Society, 84, 178, 365.
British Institute of Preventive Medicine, Transactions of the (*Review*), 355.
British Medical Benevolent Fund, Annual report of the (*Review*), 360.
Broadbent, Sir W. H. and Dr. J. F. H. —Heart disease (*Review*), 253.
Broca and F. Lubet-Barbon, Drs. A. —Mastoid abscesses and their treatment (*Review*), 260.
Brown, H.—Economics, anæsthetics, and antiseptics in the practice of midwifery (*Review*), 267.
Brown, Dr. J. J. G.—Medical diagnosis (*Review*), 250.
Browne, Dr. O. A.—Aneurysms of the aorta (*Review*), 349.
Browne, Sir T.—Religio medici, and other essays (*Review*), 328.
Brunton, Dr. T. L.—Lectures on the action of medicines (*Review*), 160.
Bushnell, Dr. F.—Thot: an exalted surgeon and physician, 123.

Caisse et de ses annexes, De l'ouverture large de la—Dr. E. J. Moure (*Review*), 77.
Campaigns, Memories of seven—Dr. J. H. Thornton (*Review*), 331.
Campbell, Dr. H.—Respiratory exercises in the treatment of disease (*Review*), 341.
Cancer of the rectum, 137.
Carcinoma by injections of mixed toxins, The treatment of sarcoma and—Dr. C. M. Moullin (*Review*), 351.
Cardiac failure—Dr. A. Morison (*Review*), 334.
Cerebral circulation, The physiology and pathology of the—Dr. L. Hill (*Review*), 150.
Childhood, The disorders of digestion in infancy and—Dr. W. S. Fenwick (*Review*), 71.
Children, Radical cure of hernia in, 135; Sarcoma of the kidney in, 132.
Children, The surgical diseases of—E. Owen (*Review*), 161.
Chloroform, Sir James Young Simpson and — H. L. Gordon (*Review*), 248.
Cholecystotomy, A case of—T. C. Grey, 311.
Cincinnati hospital, Annual report of the (*Review*), 170.
Clark, Dr. A. C.—Clinical manual of mental diseases (*Review*), 153.
Clarke, J. J.—Surgical pathology and principles (*Review*), 336.
Clarke, Dr. J. M.—Report on medicine, 314; The treatment of enteric fever, 15.
Cleland and J. Y. Mackay, Drs. J.—Human anatomy (*Review*), 148.
Clinical methods—Drs. R. Hutchison and H. Rainy (*Review*), 251.
Clinical Society of London, Transactions of the; Index to transactions (*Review*), 270.
Colera en la República Argentina, El—Dr. J. Penna (*Review*), 59.
Colitis, On a case resembling mucous —Dr. E. G. Trevithick, 120.
Coma, Differential diagnosis and treatment of—Dr. G. A. Huntley (*Review*), 349.
Conjunctival sac, Asepsis of, 53.
Coutts, Dr. J. A.—Some aspects of infantile syphilis (*Review*), 76.
Crothers and H. H. Bice, Dr. G. D. —Elements of Latin (*Review*), 346.
Curtis, Drs. H. R. Wharton and B. F.—The practice of surgery (*Review*), 254.
Cystoscopy, 238.

Dakin, Dr. W. R.—A handbook of midwifery (*Review*), 343.
Dentist, The (*Review*), 81.
Dermatology, Report on—Dr. H. Waldo, 241.
Diagnosis, Medical—Dr. J. J. G. Brown (*Review*), 250; Drs. S. and W. S. Fenwick (*Review*), 167.
Diagnosis, Practical—Dr. H. A. Hare (*Review*), 248.
Diagnosis and treatment, Surgical—Dr. J. W. Macdonald (*Review*), 337.
Diet chart and alimentary index—M. J. Kenny (*Review*), 168.
Digestion in infancy and childhood, The disorders of — Dr. W. S. Fenwick (*Review*), 71.
Diphtheria, Isolation after — Dr. B. M. H. Rogers, 101.
Doctor and patient—Dr. R. Gersuny (*Review*), 329.
Dowse, Dr. T. S. — The pocket therapist (*Review*), 168.
Dührssen, Dr. A.—A manual of obstetric practice (*Review*), 258.
Dysphagia in laryngeal tuberculosis, 322.

Ear, nose, and throat, and their accessory cavities, Diseases of the —Dr. S. S. Bishop (*Review*), 73.

Eccles, Dr. A. S.—The practice of massage: its physiological effects and therapeutic uses (*Review*), 266.

Echinodermata, The British—Dr. R. N. Wolfenden (*Review*), 262.

Edgeworth, Dr. F. H.—Hysterical paroxysmal œdema, 206; On some diseases occurring in men who worked in a sewer, 33.

Edinburgh medical journal (*Reviews*), 81, 275.

Edinburgh, Reports from the laboratory of the Royal College of Physicians (*Reviews*), 79, 353.

Ehlers, Dr. E.—Aetiologische studien über lepra (*Review*), 77.

Elder, Dr. W.—Aphasia and the cerebral speech mechanism (*Review*), 68.

Electricity, On animal—Dr. A. D. Waller (*Review*), 149.

Electricity, Practical medical—Dr. D. Turner (*Review*), 257.

Emotions, The psychology of the—T. Ribot (*Review*), 259.

Enteric fever, Complications of, 39.

Enteric fever, The treatment of—Dr. J. M. Clarke, 15.

Epidemiological Society of London, Transactions of the (*Review*), 358.

Erskine, Dr. J.—A plea for a state medical service (*Review*), 169.

Essays and addresses—Sir J. R. Reynolds (*Review*), 67.

Eye, Auto-infection of the, 54.

Eye, Diseases of the—Drs. W. F. Norris and C. A. Oliver [Eds.] (*Reviews*), 64, 162; Dr. H. R. Swanzy (*Review*), 168; Dr. D. C. Watson (*Review*), 164.

Eye, Examination of the—S. Snell (*Review*), 345.

Fenwick, Drs. S. and W. S.—The student's guide to medical diagnosis (*Review*), 167.

Fenwick, Dr. W. S.—The disorders of digestion in infancy and childhood (*Review*), 71.

Fevers and infectious diseases—Dr. W. Harding (*Review*), 270.

Fingers and toes, The deformities of the—W. Anderson (*Review*), 158.

Finlayson, Dr. J.—An account of the life and works of Dr. Robert Watt (*Review*), 74.

Firth, J. L.—A case of laparotomy in which a large pyosalpinx simulating a suppurating tubo-ovarian cyst was removed, 229.

Fisher, Dr. T.—Report on medicine, 39.

Fothergill, Dr. J. M.—The practitioner's handbook of treatment (*Review*), 256.

Fowler and R. J. Godlee, Dr. J. K.—The diseases of the lungs (*Review*), 335.

Frankland, P. and Mrs. P.—Pasteur (*Review*), 146.

Frauenheilkunde, Das studium der—A. Mackenrodt (*Review*), 352.

Gall-bladder and bile-ducts, Diseases of the—A. W. M. Robson (*Review*), 157.

Gebärmutter-scheidenkrebses, Radical-behandlung des — Dr. G. Gellhorn (*Review*), 352.

Geburtsstörungen, Die anatomie und behandlung der—Dr. W. Rühl (*Review*), 351.

Gellhorn, Dr. G.—Radical-behandlung des gebärmutter-scheidenkrebses (*Review*), 352.

Genito-urinary organs, Tuberculosis of the—Dr. N. Senn (*Review*), 338.

Gersuny, Dr. R. — Doctor and patient: hints to both (*Review*), 329.

Giles, J. B. Sutton and Dr. A. E.—Diseases of women (*Review*), 160.

Glaister, Dr. J.—A manual of hygiene for students and nurses (*Review*), 268.

Glaucoma, 52.

Godlee, Dr. J. K. Fowler and R. J.—The diseases of the lungs (*Review*), 335.

Gordon, H. L.—Sir James Young Simpson and chloroform (*Review*), 248.

Gould and W. L. Pyle, Drs. G. M.—Anomalies and curiosities of medicine (*Review*), 55.

Grant College Medical Society, Transactions of the (*Review*), 172.

Graves's disease, Treatment of—Dr. W. M. Semple, 114.

Gray, Dr. W.—Influenza (*Review*), 75.

Grey, T. C.—A case of cholecystotomy, 311.

Gumprecht, Dr. F.—Die technik der speziellen therapie (*Review*), 341.

Gynæcology in Berlin—Dr. H. Macnaughton-Jones (*Review*), 168.

Hall, Dr. A. J.—Physiology. Students' note-book (for the laboratory). Part I.—Physiological chemistry (*Review*), 167.
Hall, Dr. F. de H.—The medical examination for life assurance (*Review*), 348.
Hand, Nervous affections of the—Dr. G. V. Poore (*Review*), 264.
Harding, Dr. W.—Fevers and infectious diseases (*Review*), 270.
Hare, Dr. H. A.—Practical diagnosis (*Review*), 248.
Harsant, W. H.—Report on otology, 325; Report on surgery, 238.
Harvey, William—D'A. Power (*Review*), 145.
Health, The laboratory text-book of public—Dr. W. R. Smith (*Review*), 165.
Heart and aorta, Diseases of the—Dr. G. W. Balfour (*Review*), 333.
Heart disease—Sir W. H. and Dr. J. F. H. Broadbent (*Review*), 253.
Hedley, Dr. W. S.—Practical muscle testing and the treatment of muscular atrophies (*Review*), 257.
Herman, Dr. G. E.—Diseases of women (*Review*), 344.
Hernia in children, Radical cure of, 135.
Hernia, The radical cure of reducible—J. L. Thomas, 307.
Hewer, Mrs. L.—Our baby (*Review*), 168.
Hill, Dr. L.—The physiology and pathology of the cerebral circulation (*Review*), 150.
Holocaine, 53.
Holthouse, E.—Convergent strabismus and its treatment (*Review*), 259.
Hospital annual, Burdett's (*Review*), 360.
Hospitals, State-aided *v.* voluntary—Dr. W. K. Sibley (*Review*), 347.
Hunter, John—S. Paget (*Review*), 57.
Huntley, Dr. G. A.—Differential diagnosis and treatment of coma (*Review*), 349.
Hunyadi János—Dr. E. Monin (*Review*), 351.
Hutchison and H. Rainy, Drs. R.—Clinical methods (*Review*), 251.
Hygiene, A manual of—Dr. J. Glaister (*Review*), 268.
Hygiene and public health—Dr. L. C. Parkes (*Review*), 268.
Hygiene, Rural—Dr. H. B. Bashore (*Review*), 353.
Hysterical paroxysmal œdema—Dr. F. H. Edgeworth, 260.

Illustrirte rundschau der medicinisch-chirurgischen technik (*Review*), 361.
Index-catalogue of the library of the Surgeon-General's Office, United States Army (*Review*), 175.
Infantile alimentary canal, Septic conditions of the—Dr. F. W. F. Ross (*Review*), 71.
Infectious diseases, Fevers and—Dr. W. Harding (*Review*), 270.
Inflammation of the bladder and urinary fever—Dr. C. M. Moullin (*Review*), 339.
Influenza—Dr. W. Gray (*Review*), 75.
Intussusception, Some points in the anatomy, pathology, and surgery of—D'A. Power (*Review*), 340.
Ireland, Transactions of the Royal Academy of Medicine in (*Reviews*), 79, 356.
Isle of Man official guide, The (*Review*), 175.

Jaeger, Dr. G.—Problems of nature (*Review*), 75.
Jahresbericht über die fortschritte auf dem gebiete der chirurgie (*Review*), 70.
Janus (*Review*), 361.
Jayne, Dr. H.—Mammalian anatomy. Part I.—The skeleton of the cat (*Review*), 332.
Johns Hopkins hospital reports (*Review*), 354.

Kanthack, Drs. H. D. Rolleston and A. A.—Manual of practical morbid anatomy (*Review*), 73.
Kenny, M. J.—Diet chart and alimentary index (*Review*), 168.
Kentucky State Medical Society, Transactions of the (*Review*), 79.
Kidney in children, Sarcoma of the, 132.
King's College hospital reports (*Review*), 170.

Laparotomy, A case of—J. L. Firth, 229.
Laryngeal tuberculosis, 322.
Laryngitis, Singers', 322.
Laryngocele, Ventricular, 324.
Laryngology and rhinology, Report on—Dr. B. J. Baron, 322.
Latin, Elements of—Dr. G. D. Crothers and H. H. Bice (*Review*), 346.

Lawrie, Dr. J. M.—Notes on thirty-two consecutive abdominal sections, with thirty recoveries, performed within the last 17 months: with observations (*Review*), 350.
Lefert, P.—Lexique-formulaire des nouveautés médicales et biologiques (*Review*), 348.
Lepra, Aetiologische studien über—Dr. E. Ehlers (*Review*), 77.
Letts's medical diary (*Review*), 363.
Lewers, Dr. A. H. N.—A practical text-book on diseases of women (*Review*), 352.
Lewis, Dr. P. G.—The relief and cure of spinal curvatures (*Review*), 158.
Lewis's medical and scientific library, Catalogue of (*Review*), 360.
Lexique-formulaire des nouveautés médicales et biologiques—P. Lefert (*Review*), 348.
Library of the Bristol Medico-Chirurgical Society, 90, 186, 279, 369.
Library of the Surgeon-General's Office, United States Army, Index-catalogue of the (*Review*), 175.
Lichen scrophulosorum, 243.
Life assurance, The medical examination for—Dr. F. de H. Hall (*Review*), 348.
Local medical notes, 94, 193, 282, 378.
London, Transactions of the Clinical Society of; Index to vols. i.—xxx. of transactions (*Review*), 270.
London, Transactions of the Epidemiological Society of (*Review*), 358.
London, Transactions of the Medical Society of (*Review*), 270.
London, Transactions of the Obstetrical Society of (*Review*), 172.
Loomis and W. G. Thompson, Drs. A. L. [Eds.]—A system of medicine by American authors (*Reviews*), 60, 151.
Lubet-Barbon, Drs. A. Broca and F.—Mastoid abscesses and their treatment (*Review*), 260.
Lungs, The diseases of the—Dr. J. K. Fowler and R. J. Godlee (*Review*), 335.
Lupus, The treatment of—Dr. B. Squire (*Review*), 169.

Mac Cormac, Sir W.—Operation rooms, past and present, 301.
Macdonald, Dr. J. W.—A clinical text-book of surgical diagnosis and treatment (*Review*), 337.
Mackay, Drs. J. Cleland and J. Y.—Human anatomy (*Review*), 148.
Mackenrodt, A.—Das studium der frauenheilkunde (*Review*), 352.
McKay, Dr. W. J. S.—Lawson Tait's perineal operations, and an essay on curettage of the uterus (*Review*), 267.
Macnaughton-Jones, Dr. H.—Gynæcology in Berlin (*Review*), 168; Practical manual of diseases of women and uterine therapeutics (*Review*), 76.
Mahlendorff, P.—The science and art of adjustment between the producing and reflecting vocal apparatus (*Review*), 268.
Malaria in connection with meteorological conditions at Sierra Leone—E. M. Wilson (*Review*), 167.
Mallory and J. H. Wright, Drs. F. B.—Pathological technique (*Review*), 342.
Marcet, Dr. W.—A contribution to the history of the respiration of man (*Review*), 60.
Marshall, Dr. C. F.—Elementary physiology for nurses (*Review*), 263.
Marshall, Obituary notice of Dr. H., 193.
Massage, The practice of—Dr. A. S. Eccles (*Review*), 266.
Mastoid abscesses and their treatment—Drs. A. Broca and F. Lubet-Barbon (*Review*), 260.
Maxillaire chez les enfants, Empyème du sinus—Dr. E. J. Moure (*Review*), 77.
Maxillary antrum, Primary epithelioma of the, 324.
Medical annual (*Review*), 174.
Medicine, A system of—Dr. T. C. Allbutt [Ed.], 62; By American authors—Drs. A. L. Loomis and W. G. Thompson [Eds.] (*Reviews*), 60, 151.
Medicine, Reports on—Dr. J. M. Clarke, 314; Dr. T. Fisher, 39; Dr. G. Parker, 126; Dr. R. S. Smith, 234.
Medicine, The practice of—Dr. J. M. Anders (*Review*), 251.
Medicines, Lectures on the action of—Dr. T. L. Brunton (*Review*), 160.
Medico-chirurgical transactions (*Review*), 271.
Melancholia—Dr. L. A. Weatherly, 197.
Mental diseases—Dr. A. C. Clark (*Review*), 153.

Mexico—Memoria de los trabajos ejecutados por el Consejo Superior de Salubridad (*Review*), 269.
Michigan State Medical Society, Transactions of the (*Review*), 272.
Midwifery, A handbook of—Dr. W R. Dakin (*Review*), 343.
Midwifery, Economics, anæsthetics, and antiseptics in the practice of —H. Brown (*Review*), 267.
Midwives' pocket-book, The—Honnor Morten (*Review*), 267.
Milk and milk products, The analysis of—T. H. Pearmain and C. G. Moor (*Review*), 166.
Monin, Dr. E.—Hunyadi János (*Review*), 351.
Monsarrat, Dr. K. W.—Surgical technics in hospital practice (*Review*), 350.
Moods—F. Phillips (*Review*), 75.
Moor, T. H. Pearmain and C. G.—The analysis of food and drugs. Part I.—Milk and milk products (*Review*), 166.
Morbid anatomy, Manual of practical—Drs. H. D. Rolleston and A. A. Kanthack (*Review*), 73.
Morison, Dr. A.—On cardiac failure and its treatment (*Review*), 334.
Morris, M.—Ringworm in the light of recent research (*Review*), 346.
Morten, Honnor—The midwives' pocket-book (*Review*), 267.
Morton, C. A.—Report on surgery, 132.
Moullin, Dr. C. M.—Inflammation of the bladder and urinary fever (*Review*), 339; The treatment of sarcoma and carcinoma by injections of mixed toxins (*Review*), 351.
Moure, Dr. E. J.—De l'ouverture large de la caisse et de ses annexes; Empyème du sinus maxillaire chez les enfants; Pathogénie et traitement des déviations et éperons de la cloison du nez chez les jeunes enfants; Résultats de l'examen histologique de 64 végétations adénoïdes; Traitement de l'ozène (*Reviews*), 77; Des adénoïdites chez les adultes; Sur le traitement des sinusites (maxillaire excepté); Sur trois cas de complications intra-craniennes d'origine otique (*Reviews*), 353.
Muir and J. Ritchie, Drs. R.—Manual of bacteriology (*Review*), 165.
Muscle testing, Practical—Dr. W. S. Hedley (*Review*), 257.

Nature, Human: Its principles, and the principles of physiognomy—"Physicist" (*Review*), 262.
Nature, Problems of—Dr. G. Jaeger (*Review*), 75.
Nervensystems, Die färbetechnik des—Dr. B. Pollack (*Review*), 74.
Nervous affections of the hand—Dr. G. V. Poore (*Review*), 264.
Nervous system, Diseases of the—Dr. C. E. Beevor (*Review*), 337.
New Hampshire Medical Society, Transactions of the (*Review*), 272.
New York Academy of Medicine, Transactions of the (*Review*), 171.
New York, Transactions of the Medical Society of the State of (*Review*), 172.
Nez chez les jeunes enfants, Des déviations et éperons de la cloison du—Dr. E. J. Moure (*Review*), 77.
Norris and C. A. Oliver, Drs. W. F. [Eds.]—System of diseases of the eye (*Reviews*), 64, 162.
Nose and pharynx, Diseases of the —Dr. J. B. Ball (*Review*), 169.
Nurses, Elementary physiology for —Dr. C. F. Marshall (*Review*), 263.
Nursing directory, Burdett's official (*Review*), 81.

Obituary: Dr. A. G. Blomfield, 194; Dr. H. Marshall, 193; A. Prichard, 1.
Obstetric practice, A manual of—Dr. A. Dührssen (*Review*), 258.
Obstetrical Society of London, Transactions of the (*Review*), 172.
Obstetrics, Report on—Dr. W. C. Swayne, 139.
Occipital presentations, On—Dr. J. G. Swayne, 108.
Œdema, Hysterical paroxysmal—Dr. F. H. Edgeworth, 206.
Ohio State Medical Society, Transactions of the (*Review*), 356.
Oliver, Drs. W. F. Norris and C. A. [Eds.]—System of diseases of the eye (*Reviews*), 64, 162.
Operation rooms, past and present —Sir W. MacCormac, 301.
Ophthalmic instruments, Sterilisation of, 53.
Ophthalmological Society of the United Kingdom, Transactions of the (*Review*), 358.
Ophthalmology, Report on—C. H. Walker, 50.

Oppenheimer's ephemeris pharmacologica (*Review*), 363.
Orthoform, 54.
Osteo-myelitis of the vertebræ, Acute primary, 136.
Otique, Sur trois cas de complications intra-craniennes d'origine—Dr. E. J. Moure (*Review*), 353.
Otology, Report on—W. H. Harsant, 325.
Ovarian tumours, in pregnancy, 139; in the puerperal state, 144.
Owen, E.—The surgical diseases of children (*Review*), 161.
Oxygen treatment for wounds, ulcers, [etc.]—G. Stoker (*Review*) 266.
Ozæna, 323.
Ozène, Traitement de l'—Dr. E. J. Moure (*Review*), 77.

Paget, S.—John Hunter (*Review*), 57; Ambroise Paré and his Times (*Review*), 246.
Paré, Ambroise—S. Paget (*Review*), 246.
Parker, Dr. G.—Report on medicine, 126.
Parkes, Dr. L. C.—Hygiene and public health (*Review*), 268.
Pasteur—P. and Mrs. P. Frankland (*Review*), 146.
Patella, Fracture of the, 321.
Pathological technique—Drs. F. B. Mallory and J. H. Wright (*Review*), 342.
Pathology, Surgical—J. J. Clarke (*Review*), 336.
Pearmain and C. G. Moor, T. H.—The analysis of food and drugs. Part I.—Milk and milk products (*Review*), 166.
Penna, Dr. J.—El coléra en la República Argentina (*Review*), 59.
Perineal operations, Lawson Tait's—Dr. W. J. S. McKay (*Review*), 267.
Peritoneal drainage, Intra-, 318.
Pharmacopœia, The British (*Review*), 149; Guides to (*Reviews*), 265.
Pharynx, Diseases of the nose and—Dr. J. B. Ball (*Review*), 169.
Philadelphia County Medical Society, Transactions of the (*Review*), 273.
Philadelphia medical journal (*Review*), 175.
Philadelphia, Transactions of the College of Physicians of (*Review*), 171.
Phillips, Dr. J.—Outlines of the diseases of women (*Review*), 352.
Phillips, F.—Moods: their mental and physical character (*Review*), 75.
Phthisis, 126, 234.
"Physicist"—Human nature: its principles and the principles of physiognomy (*Review*), 262.
Physiological chemistry—Dr. A. J. Hall (*Review*), 167.
Physiology, Exercises in practical—Dr. A. D. Waller (*Review*), 348.
Physiology for nurses, Elementary—Dr. C. F. Marshall (*Review*), 263.
Physiology, Lectures on—Dr. A. D. Waller (*Review*), 149.
Plymouth Medical Society, 89.
Pneumonia, 130.
Pollack, Dr. B.—Die färbetechnik des nervensystems (*Review*), 74.
Polyneuritis in relation to gestation and the puerperium—Dr. H. G. Turney (*Review*), 352.
Poore, Dr. G. V.—Nervous affections of the hand, and other clinical studies (*Review*), 264.
Poten, Dr. W.—Die chirurgische asepsis der hände (*Review*), 76.
Pott's disease of the spine by forcible reduction, Treatment of, 45.
Power, D'A.—Some points in the anatomy, pathology, and surgery of intussusception (*Review*), 340; William Harvey (*Review*), 145.
Practice, Some incidents in general—A. Prichard (*Review*), 347.
Pregnancy, Ovarian tumours in, 139.
Prichard, A.—Some incidents in general practice (*Review*), 347.
Prichard, Augustin (In Memoriam), 1.
Progress and practice—Dr. R. Roxburgh, 285.
Psychology of the emotions, The—T. Ribot (*Review*), 259.
Public health (*Review*), 362.
Puerperal state, Ovarian tumours in the, 144.
Pyle, Drs. G. M. Gould and W. L.—Anomalies and curiosities of medicine (*Review*), 55.

Rabagliati, Dr. A.—Air, food, and exercises (*Review*), 264.
Rainy, Drs. R. Hutchison and H.—Clinical methods (*Review*), 251.
Rectum, Cancer of the, 137.
Religio medici—Sir T. Browne (*Review*), 328.
Respiration of man—Dr. W. Marcet (*Review*), 60.
Respiratory exercises–Dr. H. Campbell (*Review*), 341.

"Review of Reviews," The medical and surgical (*Review*), 361.
Reynolds, Sir J. R.—Essays and addresses (*Review*), 67.
Reynolds, S. H.—The vertebrate skeleton (*Review*), 149.
Rhinology, Report on laryngology and—Dr. B. J. Baron, 322.
Ribot, T.—The psychology of the emotions (*Review*), 259.
Ringer and H. Sainsbury, Drs. S.—A handbook of therapeutics (*Review*), 255.
Ringworm and alopecia areata—Dr. H. Aldersmith (*Review*), 260.
Ringworm — M. Morris (*Review*), 346.
Ritchie, Drs. R. Muir and J.—Manual of bacteriology (*Review*), 165.
Roberts-Jones, M.—Handbook on the workmen's compensation act, 1897 (*Review*), 359.
Robson, A. W. M.—Diseases of the gall-bladder and bile-ducts (*Review*), 157.
Röntgen rays in laryngology and rhinology, 324; Röntgen rays in ophthalmology, 50; Radiography in marine zoology—Dr. R. N. Wolfenden (*Review*), 262.
Rogers, Dr. B. M. H.—Isolation after diphtheria, 101.
Rolleston and A. A. Kanthack, Drs. H.D.—Manual of practical morbid anatomy (*Review*), 73.
Ross, Dr. F. W. F.—Septic conditions of the infantile alimentary canal, and their treatment (*Review*), 71.
Roxburgh, Dr. R.—Progress and practice, 285.
Rühl, Dr. W.—Die anatomie und behandlung der geburtsstörungen (*Review*), 351.

Sainsbury, Drs. S. Ringer and H.—A handbook of therapeutics (*Review*), 255.
Saint Bartholomew's hospital reports (*Review*), 170.
St. Thomas's hospital reports (*Review*), 355.
Sanitary inspector's handbook, The —A. Taylor, 268.
Sarcoma and carcinoma by injections of mixed toxins, The treatment of — Dr. C. M. Moullin (*Review*), 351.
Scraps, 95, 195, 283, 381.
Semple, Dr. W. M.—A suggestion for the treatment of Graves's disease, 114; A suggested method of copying sphygmograms, 312.
Senn, Dr. N.—Tuberculosis of the genito-urinary organs (*Review*), 338.
Sewer, Diseases in men who worked in a—Dr. F. H. Edgeworth, 33.
Shadwell, Dr. A. [Ed.]—The Tallerman treatment by superheated dry air (*Review*), 350.
Shaw-Mackenzie, Dr. J. A.—On maternal syphilis, including the presence and recognition of syphilitic pelvic disease in women (*Review*), 344.
Shaw's manual of the vaccination law (*Review*), 359.
Sibley, Dr. W. K.—State aided *v.* voluntary hospitals (*Review*), 347.
Sick, Preparations for the, 82, 176, 276, 363.
Sierra Leone, Malaria in connection with meteorological conditions at —E. M. Wilson (*Review*), 167.
Simpson and chloroform, Sir James Young—H. L. Gordon (*Review*), 248.
Sinusites (maxillaire excepté), Sur le traitement des (*Review*), 353.
Sinus thrombosis, 325.
Skin, Excretory irritation, and the action of certain internal remedies on the—Dr. D. Walsh (*Review*), 169.
Smith, J. G.—Abdominal surgery (*Review*), 154.
Smith Memorial, Greig, 379.
Smith, N.—Spinal caries (*Review*), 265.
Smith, Dr. R. S.—Report on medicine, 234.
Smith, Dr. W. R.—The laboratory text-book of public health (*Review*), 165.
Smithsonian report (*Review*), 175.
Snell, S.—A practical guide to the examination of the eye (*Review*), 345.
Societies: Bristol Medico-Chirurgical, 84, 178, 365; Plymouth Medical, 89.
Sphygmograms, A suggested method of copying—Dr. W. M. Semple, 312.
Spinal caries—N. Smith (*Review*), 265.
Spinal cord, Diseases of the—Dr. B. Bramwell (*Review*), 154.
Spinal curvatures, The relief and cure of—Dr. P. G. Lewis (*Review*), 158.

Sprigge, Dr. S. S.—The life and times of Thomas Wakley (*Review*), 330.
Squire, Dr. B.—The treatment of lupus (*Review*), 169.
State medical service, A plea for a—Dr. J. Erskine (*Review*), 169.
Stethoscope, The (*Review*), 80.
Stoker, G.—The oxygen treatment for wounds, ulcers, burns, scalds, lupus, and diseases of the nose, eye, and ear (*Review*), 266.
Stomach, Diseases of the, 314.
Strabismus, Convergent—E. Holthouse (*Review*), 259.
Surgery—W. J. Walsham (*Review*), 255.
Surgery, Operative—H. J. Waring (*Review*), 338.
Surgery, Reports on—W. M. Barclay, 45; W. H. Harsant, 238; C. A. Morton, 132; Dr. J. Swain, 137, 318.
Surgery, The practice of—Drs. H. R. Wharton and B. F. Curtis (*Review*), 254.
Surgical technics in hospital practice—Dr. K. W. Monsarrat (*Review*), 350.
Sutton and Dr. A. E. Giles, J. B.—The diseases of women (*Review*), 160.
Swain, Dr. J.—Fifty consecutive intra-abdominal operations, 211; Reports on surgery, 137, 318.
Swanzy, Dr. H. R.—A handbook of the diseases of the eye (*Review*), 168.
Swayne, Dr. J. G.—On occipital presentations, 108.
Swayne, Dr. W. C.—Report on obstetrics, 139.
Syphilis, Infantile—Dr. J. A. Coutts (*Review*), 76.
Syphilis, Maternal—Dr. J. A. Shaw-Mackenzie (*Review*), 344.

Tait's perineal operations, Lawson—Dr. W. J. S. McKay (*Review*), 267.
Tallerman treatment by superheated dry air, The—Dr. A. Shadwell [Ed.] (*Review*), 350.
Taylor, A.—The sanitary inspector's handbook (*Review*), 268.
Temperature of the body in health and disease, On the—Dr. W. H. White (*Review*), 263.
Tetanus, 131.
Therapeutics, A handbook of—Drs. S. Ringer and H. Sainsbury (*Review*), 255.
Therapie, Die technik der speziellen—Dr. F. Gumprecht (*Review*), 341.
Therapist, The pocket—Dr. T. S. Dowse (*Review*), 168.
Thomas, J. L.—The radical cure of reducible hernia by Kocker's method of invagination, with lateral displacement of the neck of the sac, 307.
Thompson, Drs. A. L. Loomis and W. G. [Eds.]—A system of practical medicine by American authors (*Reviews*), 60, 151.
Thornton, Dr. J. H.—Memories of seven campaigns (*Review*), 331.
Thot: an exalted surgeon and physician—Dr. F. Bushnell, 123.
Toes, The deformities of the fingers and—W. Anderson (*Review*), 158.
Toxins, The treatment of sarcoma and carcinoma by injections of mixed—Dr. C. M. Moullin (*Review*), 351.
Treatment, The practitioner's handbook of—Dr. J. M. Fothergill (*Review*), 256.
Trevithick, Dr. E. G.—A case resembling in many respects the condition known as mucous colitis, 120.
Tuberculosis, 126, 234.
Tuberculosis, Cutaneous, 241.
Tuberculosis, Laryngeal, 322.
Tuberculosis of the genito-urinary organs—Dr. N. Senn (*Review*), 338.
Turner, Dr. D.—A manual of practical medical electricity (*Review*), 257.
Turney, Dr. H. G.—Polyneuritis in relation to gestation and the puerperium (*Review*), 352.
Typhoid fever, *see* Enteric fever.

Uterine therapeutics, Diseases of women and—Dr. H. Macnaughton-Jones (*Review*), 76.

Vaccination law, Shaw's manual of the (*Review*), 359.
Vertebræ, Acute primary osteomyelitis of the, 136.
Vertebrate skeleton, The—S. H Reynolds (*Review*), 149.
Vocal apparatus, The science and art of adjustment between the producing and reflecting—P. Mahlendorff (*Review*) 268.

Wakley, The life and times of Thomas—Dr. S. S. Sprigge (*Review*), 330.
Waldo, Dr. H.—Report on dermatology, 241.
Walker, C. H.—Report on ophthalmology, 50.
Waller, Dr. A. D.—Exercises in practical physiology (*Review*), 348; Lectures on physiology. First Series.—On animal electricity (*Review*), 149.
Walsh, Dr. D.—Excretory irritation, and the action of certain internal remedies on the skin (*Review*), 169.
Walsham, W. J.—Surgery: its theory and practice (*Review*), 255.
Waring, H. J.—Manual of operative surgery (*Review*), 338.
Watson, Dr. D. C.—Practical handbook of the diseases of the eye (*Review*), 164.
Watt, Life and works of Dr. Robert—Dr. J. Finlayson (*Review*), 74.
Weatherly, Dr. L. A.—Melancholia, 197.
Webster, Dr. J. C.—Diseases of women (*Review*), 344.
Wellcome's medical diary and visiting list (*Review*), 363.
West Indies, Yellow fever in the—Dr. I. Anderson (*Review*), 349.
Westminster hospital reports (*Review*), 171.
Wharton and B. F. Curtis, Drs. H. R.—The practice of surgery (*Review*), 254.
White, Dr. W. H.—The means by which the temperature of the body is maintained in health and disease (*Review*), 263.
Wilson, E. M.—Notes on malaria in connection with meteorological conditions at Sierra Leone (*Review*), 167.
Wolfenden, Dr. R. N.—Radiography in marine zoology. The British echinodermata (*Review*), 263.
Women, Diseases of—Dr. G. E. Herman (*Review*), 344; Dr. A. H. N. Lewers (*Review*), 352; Dr. H. Macnaughton-Jones (*Review*), 76; Dr. J. Phillips (*Review*), 352; J. B. Sutton and Dr. A. E. Giles (*Review*), 160; Dr. J. C. Webster (*Review*), 344.
Workmen's compensation act, Handbook on the—M. Roberts-Jones (*Review*), 359.
Wright, Drs. F. B. Mallory and J. H.—Pathological technique (*Review*), 342.
Wright's visiting list (*Review*), 363.

Year-book of scientific and learned societies (*Review*), 360.
Year-book of treatment (*Review*), 80.
Yellow fever in the West Indies—Dr. I. Anderson (*Review*), 349.

---

J. W. Arrowsmith, Printer, Quay Street, Bristol.

### *PUBLICATIONS RECEIVED.*

From FÉLIX ALCAN, Paris:

*Annales d'Électrobiologie.* 15 Juillet, 1898.

From THE ARLINGTON CHEMICAL COMPANY, Youkers, N.Y.:

*The Doctor's Factotum.* September, October, 1898.

From Dr. WM. B. ATKINSON, Philadelphia:

*Public Health.* October, 1898.

From BAILLIÈRE, TINDALL AND COX, London:

*Aids to Examinations.* Part II. By T. REUELL ATKINSON, M.D. [N.D.]
*How to Avoid Tubercle.* By A. T. TUCKER WISE, M.D. 1898.

From JOHN BALE, SONS & DANIELSSON, LTD., London:

*The Journal of Balneology and Climatology.* October, 1898.

From FRIEDR. BAYER & Co., Elberfeld:

*Clinical Excerpts.* September, October, 1898.

From Dr. L. DUNCAN BULKLEY, New York:

*Manifestations of Syphilis in the Mouth.* By L. DUNCAN BULKLEY, A.M., M.D. 1898. [Reprint.]
*The Dangers of Specialism in Medicine.* By L. DUNCAN BULKLEY, A.M., M.D. 1898. [Reprint.]

From BURROUGHS, WELLCOME & Co., London:

*Wellcome's Medical Diary and Visiting List.* 1899.

From CALIFORNIA MEDICAL COLLEGE, San Francisco:

*California Medical Journal.* September—November, 1898.

From CASSELL AND COMPANY, LIMITED, London:

*The Origin and Progress of Renal Surgery.* By HENRY MORRIS, M.A., M.B. 1898.
*Justus von Liebig: his Life and Work.* By W. A. SHENSTONE. 1895.
*Charles Darwin and the Theory of Natural Selection.* By EDWARD B. POULTON, M.A., F.R.S. 1896.
*Diseases of the Skin.* By MALCOLM MORRIS. New Edition. 1898.
*Letts's Medical Diary for the Year 1899.*
*Do. do. do. Interleaved.*

From J. & A. CHURCHILL, London:

*Diet and Food.* By ALEXANDER HAIG, M.A., M.D. 1898.
*A Synopsis of Surgery.* By R. F. TOBIN. 1898.
*Mental Diseases.* By T. S. CLOUSTON, M.D. Fifth Edition. 1898.
*The Practice of Medicine.* By FREDERICK TAYLOR, M.D. Fifth Edition. 1898.
*Ovariotomy and Abdominal Surgery.* By HARRISON CRIPPS. 1898.
*A Treatise on Anatomy.* Edited by HENRY MORRIS, M.A. and M.B. Lond. Second Edition. 1898.
*Ophthalmic Surgery and Medicine.* By WALTER H. H. JESSOP, M.A., M.B. 1898.
*A Manual of Bacteriology.* By RICHARD T. HEWLETT, M.D. 1898.
*Observations on Cardio-vascular Repair.* By W. BEZLY THORNE, M.D. 1898.

From CHURCH MISSIONARY SOCIETY, London:

*Mercy and Truth.* October—December, 1898.

From JAMES CLEGG, Rochdale:

*Directory of Booksellers.* Edited by JAMES CLEGG. 1899 [*sic*].

From CONAWAY & SHAW, Des Moines:

*Contributions to Orthopedic Surgery.* By JAMES W. COKENOWER, A.M., MD. 1898.

From ARCHIBALD CONSTABLE & Co., Westminster [London]:

*The Surgical Anatomy of the Lymphatic Glands.* By CECIL H. LEAF, M.A., M.B. 1898.

From THE F. A. DAVIS COMPANY, Philadelphia:

*Psychology and Mental Disease.* By C. B. BURR, M.D. Second Edition. 1898.
*Practical Uranalysis and Urinary Diagnosis.* By CHARLES W. PURDY, M.D., LL.D. Fourth Edition. 1898.

From EDUARDO DUBLAN, Mexico:

*Boletin del Consejo superior de Salubridad.* Agosto—Octubre, 1898.

From Dr. LANDON B. EDWARDS, Richmond, U.S.A.

*Virginia Medical Semi-Monthly.* August 12—November 25, 1898.

From FERET ET FILS, Bordeaux

*De la Trachéo-Thyrotomie dans le Cancer du Larynx.* Par le Dr. E J. MOURE. 1898.

From GUSTAV FISCHER, Jena:

*Ueber die Wirkung des neuen Tuberkulins TR.* Von Dr. H. STROEBE. 1898.

From HENRY J. GLAISHER, London:

*On the Hand.* By EDWARD BLAKE, M.D. [1898.]
*On So-Called Spasmodic Asthma.* By ERNEST KINGSCOTE, M.B., C.M. 1899 [*sic*].

From Drs. P. HARTENBERG & P. VALENTIN, Paris:

*Revue de Psychologie.* Sept.—Nov., 1898.

From A. HELBIG, London:

*The Therapist.* November, 1898.

From IMPRENTA DEL GOBIERNO, Mexico:

*Informes Rendidos por los Inspectores Sanitarios de Cuartel y por los de los Distritos al Consejo superior de Salubridad* [1896]. 1898.
*Informes Rendidos por los Inspectores Sanitarios de Cuartel y por los de los Distritos al Consejo superior de Salubridad. Correspondientes al año de 1897.* 1898.

From INGRAM BROTHERS, London:

*The English Illustrated Magazine.* October, 1898.

From L'INSTITUT DE BIBLIOGRAPHIE, Paris:

*Gazette médicale de Paris.* 10 Septembre—3 Décembre, 1898.
*Statistique des Opérations pratiquées au Mans, 1896.* Par H. DELAGÉNIÈRE. 1897.

From S. KARGER, Berlin:

*Die Störungen des Verdauungsapparates als Ursache und Folge anderer Erkrankungen.* Von Dr. HANS HERZ. 1898.
*Fürsorge für Gemüthskranke.* Von Prof. Dr. C. FÜRSTNER. 1899 [*sic*].

From HENRY KIMPTON, London:

*Modern Surgery.* By JOHN CHALMERS DACOSTA, M.D. [Second Edition]. 1898.
*Essentials of Diseases of the Ear.* By E. B. GLEASON, S.B., M.D. Second Edition. 1898.
*Pathogenic Bacteria.* By JOSEPH MCFARLAND, M.D. Second Edition. 1898.

From H. K. LEWIS, London:

*Materia Medica.* By WILLIAM MARTINDALE. Tenth Edition. 1898.
*Unripe Cataract.* By WILLIAM A. M'KEOWN, M.D., M.Ch. 1898.

From R. E. LYNCH, Philadelphia:

*Report of the Kensington Hospital for Women, Philadelphia, 1896-97.*

From JAMES MACLEHOSE AND SONS, Glasgow:

*Atlas of External Diseases of the Eye.* By A. MAITLAND RAMSAY, M.D. 1898.

From MACMILLAN AND CO., LIMITED, London:

*The Cold-Bath Treatment of Typhoid Fever.* By F. E. HARE, M.D. 1898.
*Diseases of the Breast.* By A. MARMADUKE SHEILD, M.B. 1898.

From THE MEDICAL PUBLISHING COMPANY, LIMITED, London:

*The Transactions of the Society of Anæsthetists.* Vol. I. 1898.

From P. A. NORSTEDT & SÖNER, Stockholm:

*Forhandlinger ved Nordisk Kirurgisk Forenings 3:je Möde.* 1898.

From OPPENHEIMER, SON & CO., LTD., London:

*Ephemeris Pharmacologica.* 1899.

From YOUNG J. PENTLAND, Edinburgh:

*Diseases of the Heart and Aorta.* By GEORGE A. GIBSON, M.D. 1898.
*Contributions to Clinical Medicine.* By T. M'CALL ANDERSON, M.D. Edited by JAMES HINSHELWOOD, M.A., M.D. 1898.

From PRICE'S PATENT CANDLE COMPANY, London:

*Glycerine.* By M.D. 1898.

From *La Rassegna Medica*, Bologna:

*Tannigeno e Tannalbina. Pel Dott. Francesco Corletto.* 1898. [Reprint.]

From THE REBMAN PUBLISHING CO., LIMITED, London:

*Archives of the Roentgen Ray.* August, 1898.

From THE SCIENTIFIC PRESS, LIMITED, London:

*An Atlas of Bacteriology.* By CHAS. SLATER, M.A., M.B., and EDMUND J. SPITAT. 1889.

From SHAW & SONS, London:

*Vaccination Law.* By a Barrister-at-Law. Sixth Edition. 1898.

From SMITH, ELDER & CO., London:

*Traumatic Separation of the Epiphyses.* By JOHN POLAND. 1898.
*The Science and Practice of Midwifery.* By W. S. PLAYFAIR, M.D. Ninth Edition. 1898.

From THE SMITHSONIAN INSTITUTION, Washington:

*An Investigation on the Influence upon the Vital Resistance of Animals to the Micro-organisms of Disease brought about by Prolonged Sojourn in an Impure Atmosphere.* By D. H. BERGEY, M.D. 1898.

From THE "TATVA-VIVECHAKA" PRESS, Bombay:

*Transactions of the Grant College Medical Society, 1897.* 1898.

From WATTS & CO., London:

*Registration of Midwives.* By THOMAS M. WATT, M.A. 1898.

From WILLIAMS AND NORGATE, London:

*Janus.* Juillet—Août, 1898.

From HUGH M. WILSON, Chicago:

*The Railway Surgeon.* September 6—November 15, 1898.

From JOHN WRIGHT & CO., Bristol:

*Wright's Improved Physicians', Surgeons', and Consultants' Visiting List.* 1899.
*Do. do. do. Perpetual.*

From K. J. WYSS, Berne:

*Illustrirte Rundschau der medicinisch-chirurgischen Technik.* November, 1898.

---

### Bristol Medico-Chirurgical Journal.

Terms for Advertisements.

| | £ | s. | d. |
|---|---|---|---|
| Page ... ... ... ... ... ... ... ... ... ... ... ... ... | 1 | 1 | 0 |
| Half Page ... ... ... ... ... ... ... ... ... ... ... ... | 0 | 12 | 6 |
| Quarter Page ... ... ... ... ... ... ... ... ... ... ... | 0 | 7 | 6 |

Insets (2 or 4 pages), 20/-

Upon orders given for four consecutive insertions, a discount of 25 per cent. will be allowed, if payment is made within one month from date of first insertion. Terms for special positions upon application.

*Orders to be sent to the Editorial Secretary,* B. ROGERS, M.D., *Clifton, Bristol.*

